# Supplementary material for: Graphite phase carbon nitride based membrane for selective permeation
Source: Nat Commun. 2019 Jun 7;10:2500. doi: 10.1038/s41467-019-10381-z (PMC6555826; doi:10.1038/s41467-019-10381-z)
Supplement: Supplementary file 1 — Supplementary information [file 41467_2019_10381_MOESM1_ESM.pdf]

Graphite phase carbon nitride based membrane for selective permeation

Wang et al.

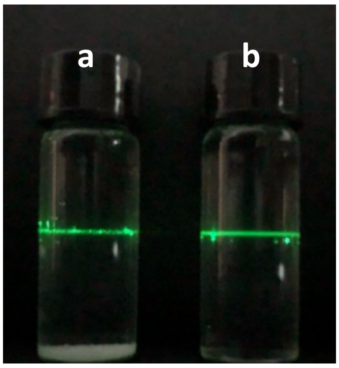

Supplementary Figure 1 | Digital photos of nanosheet suspension with Tyndall effect. a, b, GCN (**a**) and GCN-SA (**b**) nanosheet suspensions (both at 10 mg L^{-1}) after free standing for six months.

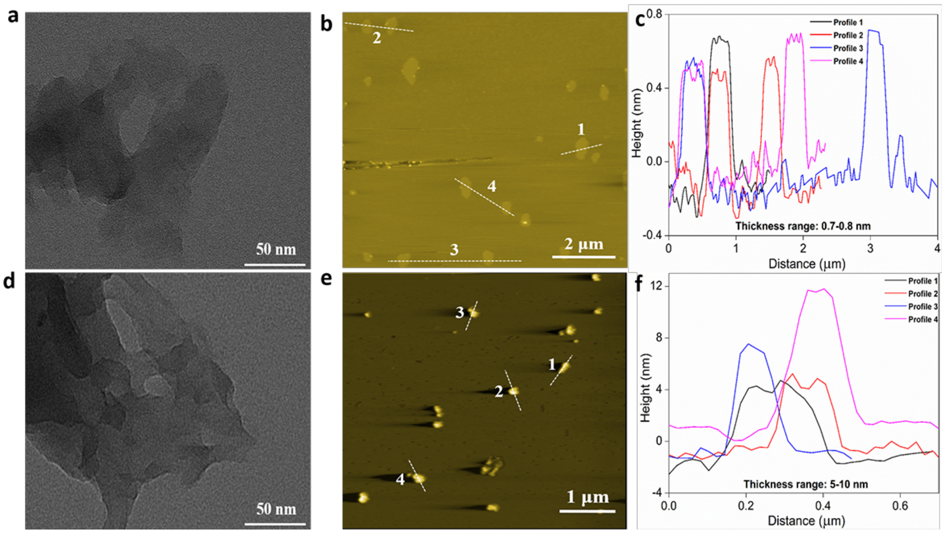

Supplementary Figure 2 | Microstructure of GCN and GCN-SA nanosheets. a, b, c, TEM image (**a**), AFM image (**b**) and corresponding height profile (**c**) of GCN nanosheets. **d, e, f,** TEM image (**d**), AFM image (**e**) and corresponding height profile (**f**) of GCN-SA nanosheets.

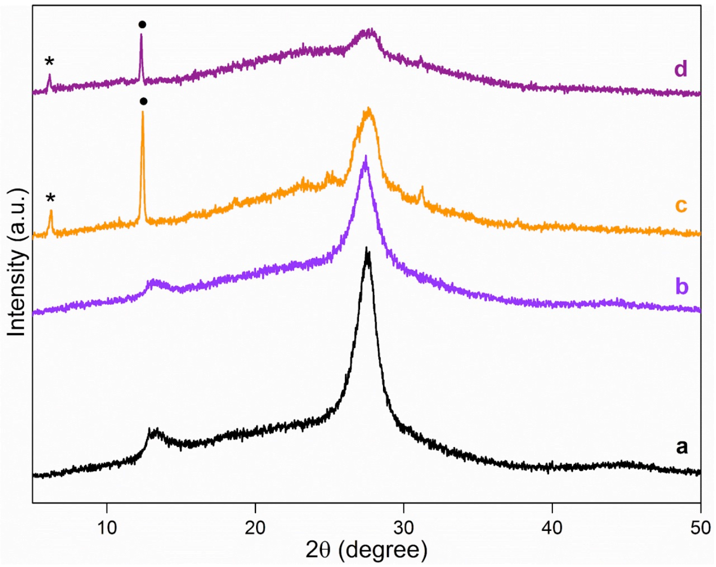

Supplementary Figure 3 | XRD patterns of GCN and GCN-SA composite. a, Bulk GCN for reference. **b**, GCN nanosheets dried at 80 °C under vacuum. **c, d,** GCN-SA nanosheets dried at room temperature (**c**) and re-immersed in water (**d**). New diffraction peaks (001n) and (002n) are marked as black stars and balls, respectively, n represents the new phase.

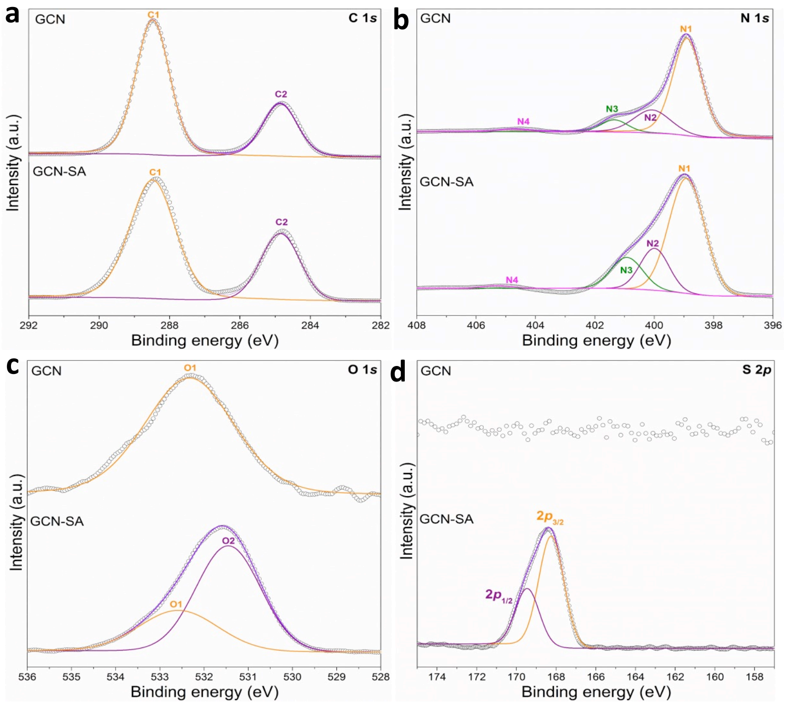

Supplementary Figure 4 | High resolution XPS spectra of GCN and GCN-SA nanosheets. a, C 1*s*. **b**, N 1*s*. **c**, O 1*s*. **d**, S 2*p*. All the dotted black lines represent the raw XPS data.

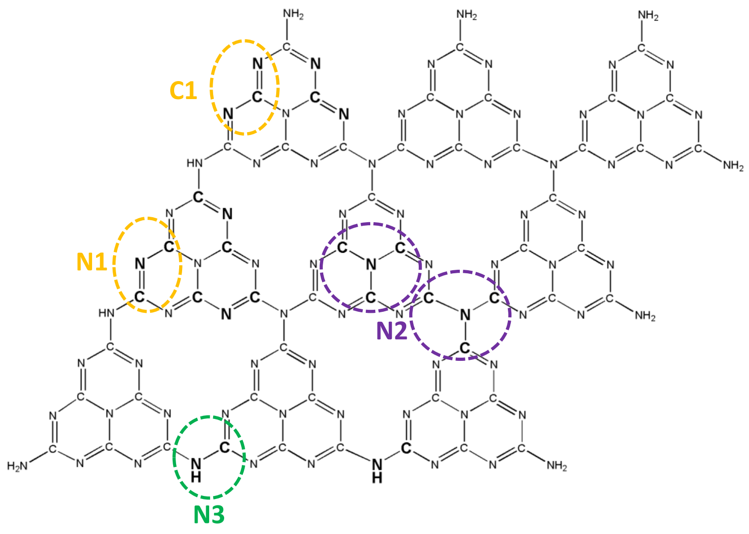

Supplementary Figure 5 | Representative bonding characteristics of GCN. The dotted orange circles denote N-C=N bonding (C1) and C-N=C bonding (N1), the dotted violet circles denote $N\text{-}(C)_3$ bonding (N2), the dotted blue circle denotes C-N-H bonding (N3).

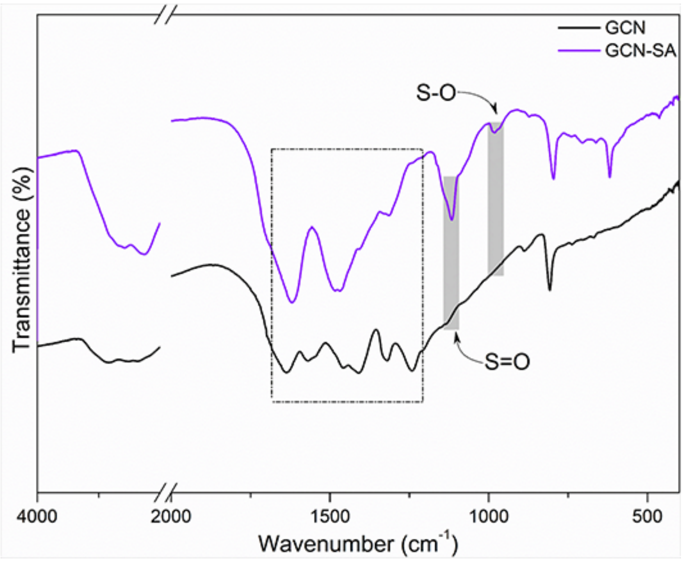

Supplementary Figure 6 | FT-IR spectra of GCN and GCN-SA nanosheets. The dotted black rectangle within 1200-1700 cm^{-1} denotes the characteristic C-N and C=N vibrations of GCN.

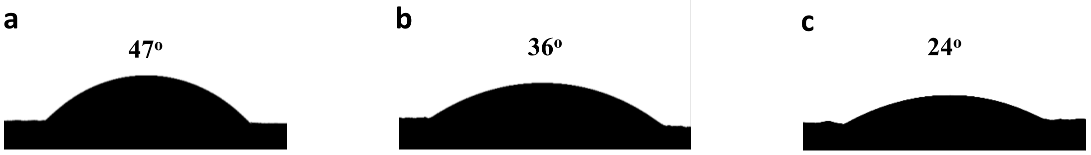

Supplementary Figure 7 | Digital photos of water drop on membranes. a, GCN. **b,** GCN-CSA. **c,** GCN-SA.

The contact angle of GCN-SA membrane has been discussed in main text. With respect to GCN membrane, protonation of GCN by CSA increased its hydrophilicity. On the other hand, organic group on CSA entitles GCN-CSA higher hydrophobicity than GCN-SA. This agrees with the contact angle measurement as displayed in Supplementary Figure 7. The contact angle of GCN-CSA membrane to water (36°) is lower than that of GCN membrane (47°) but higher than that of GCN-SA membrane (24°).

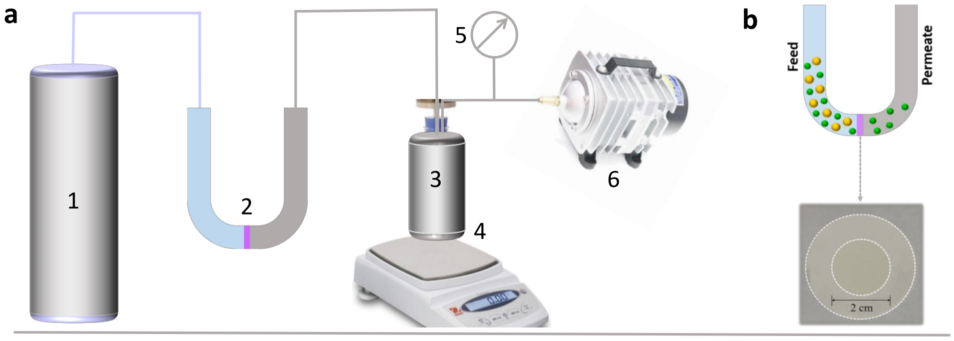

1—solvent reservoir; 2—U-shaped device; 3—sealed bottle; 4—electronic balance; 5—pressure gage; 6—air pump.

Supplementary Figure 8 | Devices for solvent permeability and permeation tests. **a,** Home-made device for solvent permeability test. The membrane glued on silicon pad is marked by violet color. **b,** U-shaped device for permeation test (component 2 in **a**) and the digital image of membrane on substrate. The dotted violet arrow aims to show the digital photo of membrane that is installed in U-shaped device.

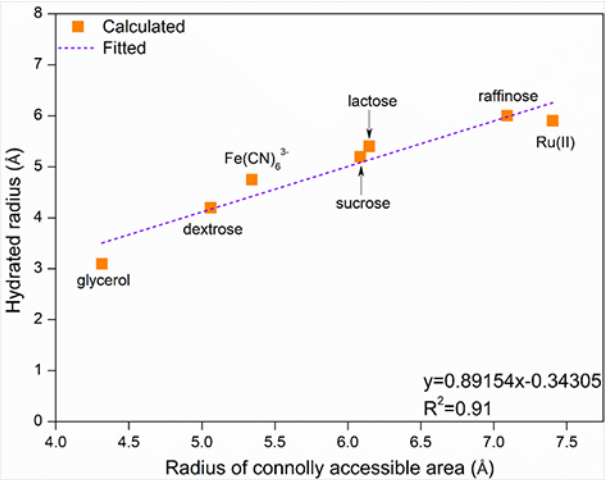

Supplementary Figure 9 | Correlation between hydrated radius and CAA radius. Ru(II) represents tris-(bipyridine)-runthenium (II) chloride. The dotted violet line denotes the linear fitting result of hydrated radius which varies as a function of CAA radius.

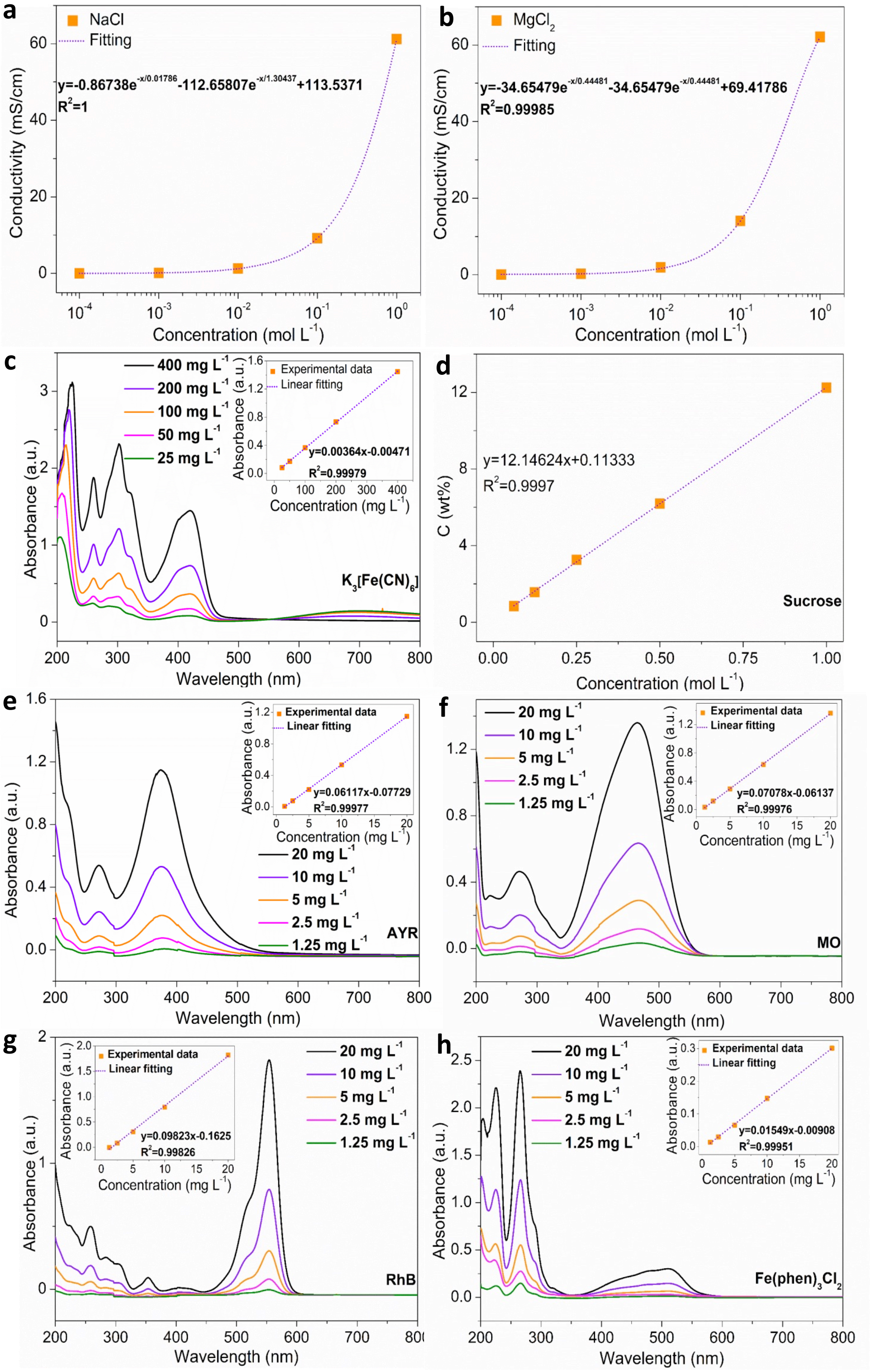

Supplementary Figure 10 | Standard curves of solutes in aqueous solution. The dotted violet lines denote the fitting results of solute absorbance. **a,** NaCl. **b,** $MgCl_2$. **c,** sucrose. **d,** $K_3[Fe(CN)_6]$. **e,** AYR. **f,** MO. **g,** RhB. **h,** $Fe(phen)_3Cl_2$.

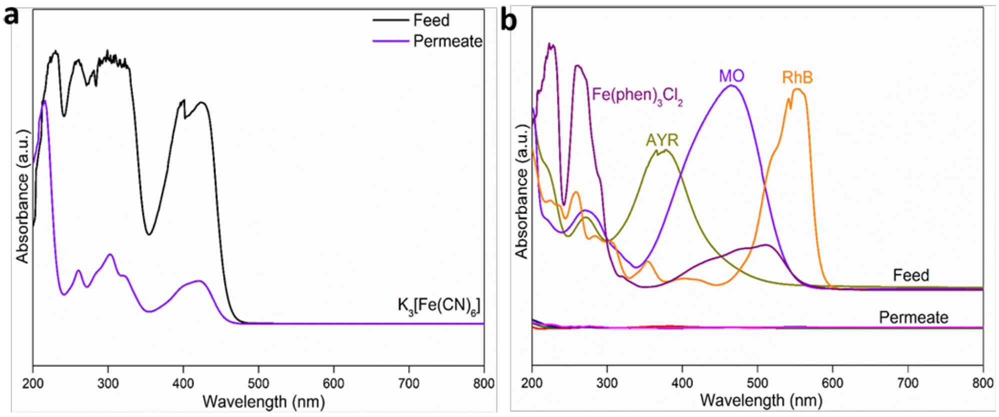

Supplementary Figure 11 | UV-Vis absorption spectra of feed and permeate solutions. a, $K_3[Fe(CN)_6]$. **b,** $Fe(phen)_3Cl_2$, AYR, MO and RhB.

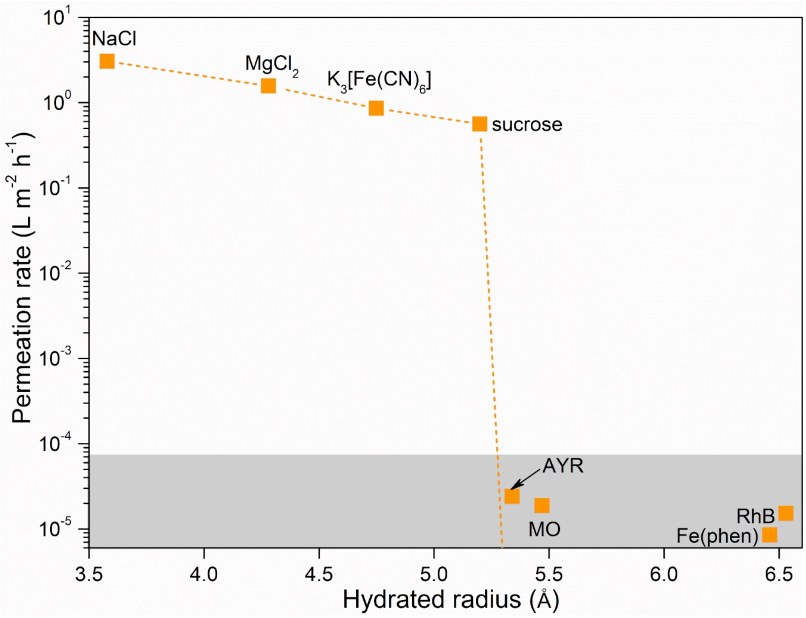

Supplementary Figure 12 | Permeation of different solutes through GCN-SA membranes. Sieving performance of varied solutes with the same initial concentration of 0.005 M through 700 nm-thick GCN-SA membrane. The permeation rates of all solutes are calculated according to Equation 3 in Methods section of main text. The dotted orange line denotes the permeation rates of solutes with different hydrated radii. The scattered points fall in the grey area represent the detection limit.

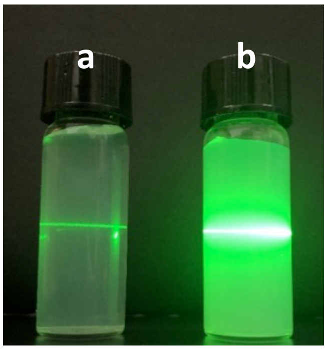

Supplementary Figure 13 | Digital photos of nanosheet dispersion with Tyndall effect. a, b, GCN for comparison (**a**) and GCN-CSA (**b**) fabricated by ball milling method and further centrifugation at 12000 rpm for 5 min to remove large particles.

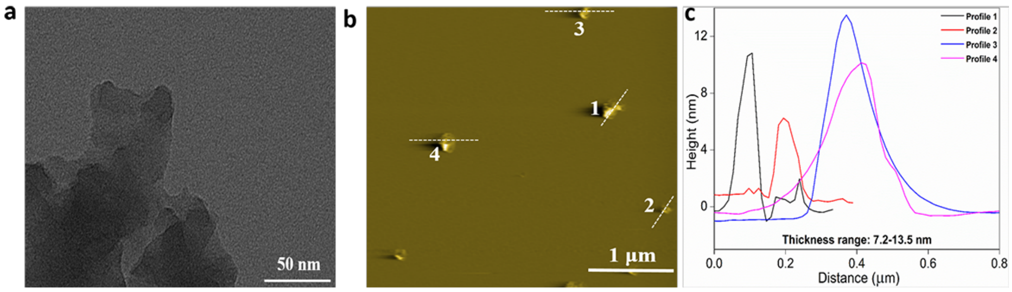

Supplementary Figure 14 | Microstructure of GCN-CSA nanosheets. a, b, c, TEM image (**a**), AFM image (**b**) and corresponding height profile (**c**) of GCN-CSA nanosheets.

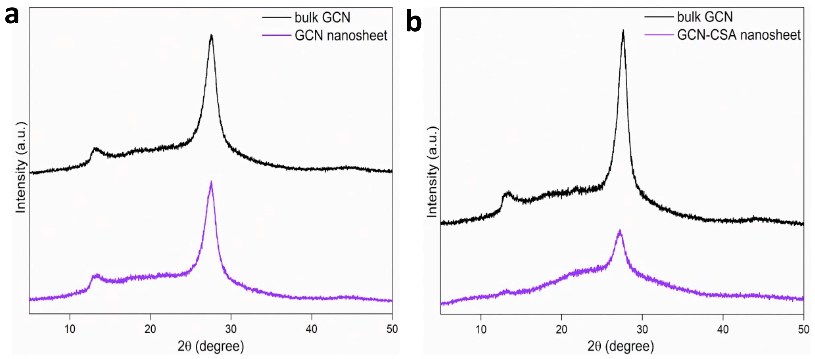

Supplementary Figure 15 | XRD patterns of ball-milled GCN and GCN-CSA samples. a, Pristine bulk GCN and ball-milled GCN nanosheets. **b,** Pristine bulk GCN and ball-milled GCN-CSA nanosheets.

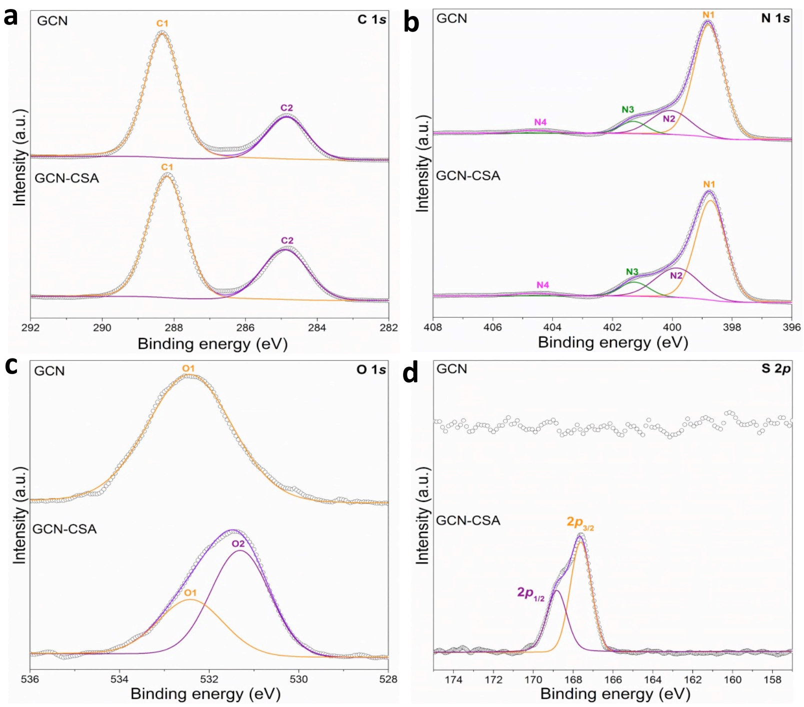

Supplementary Figure 16 | High resolution XPS spectra of GCN and GCN-CSA nanosheets. a, C 1*s*. **b**, N 1*s*. **c**, O 1*s*. **d**, S 2*p*. All dotted black lines denote the raw XPS data.

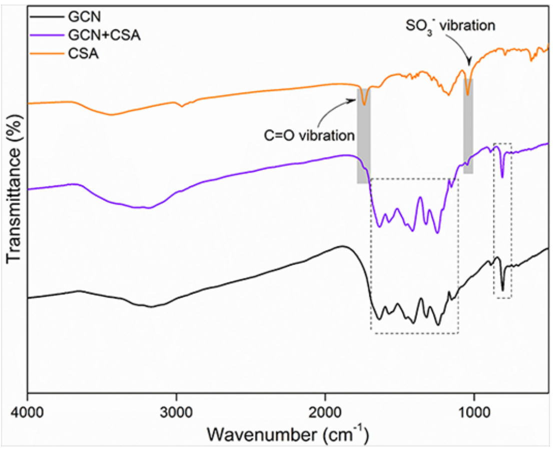

Supplementary Figure 17 | FT-IR spectra of GCN, GCN-CSA and CSA. The dotted black rectangles within 1200-1700 cm^{-1} and ~810 cm^{-1} denote the characteristic stretching and bending vibrations of GCN, respectively.

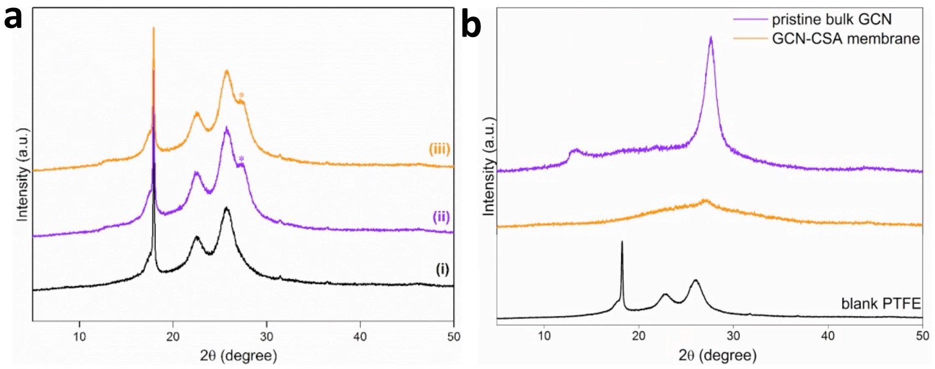

Supplementary Figure 18 | XRD patterns of GCN-CSA membranes. a, Blank PTFE (i), dried GCN-CSA membrane on PTFE (ii) and dried GCN-CSA membrane on PTFE after immersing in water for 3 days (iii). The characteristic (002) peaks of GCN are marked as violet or orange star. **b,** XRD patterns of pristine bulk GCN and GCN-CSA membrane without PTFE support (XRD pattern of blank PTFE is also provided for comparison).

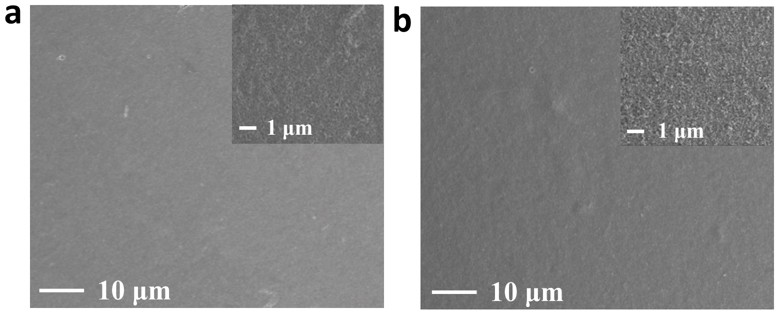

Supplementary Figure 19 | Top-viewed SEM images of as-prepared membranes. **a,** GCN. **b,** GCN-CSA. The inserts show the SEM images at higher magnification.

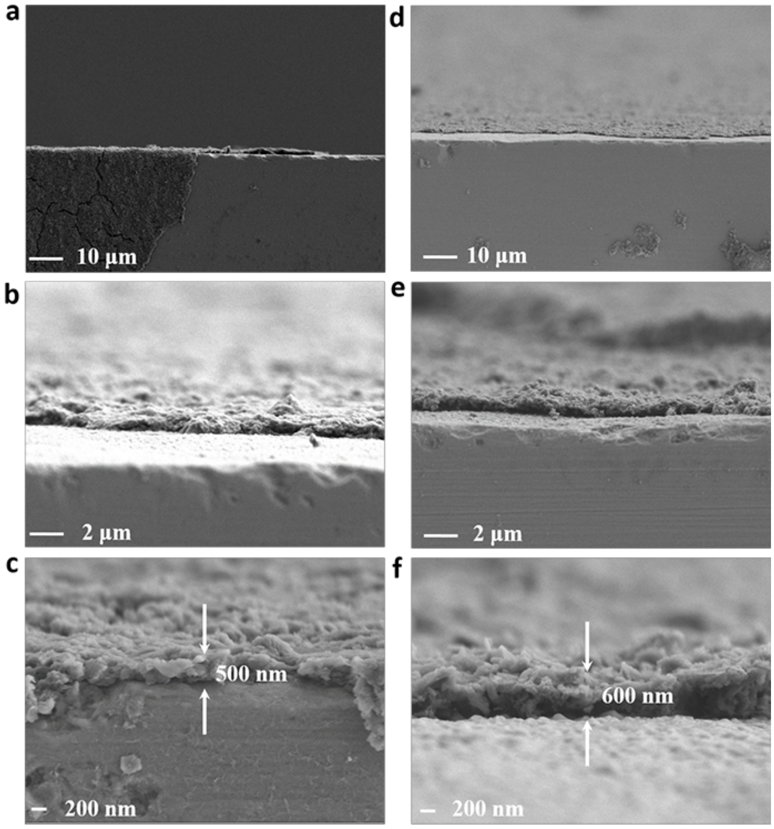

Supplementary Figure 20 | Cross-sectional SEM images of membranes at different magnification. a, b, c, GCN membrane. **d, e, f,** GCN-CSA membrane.

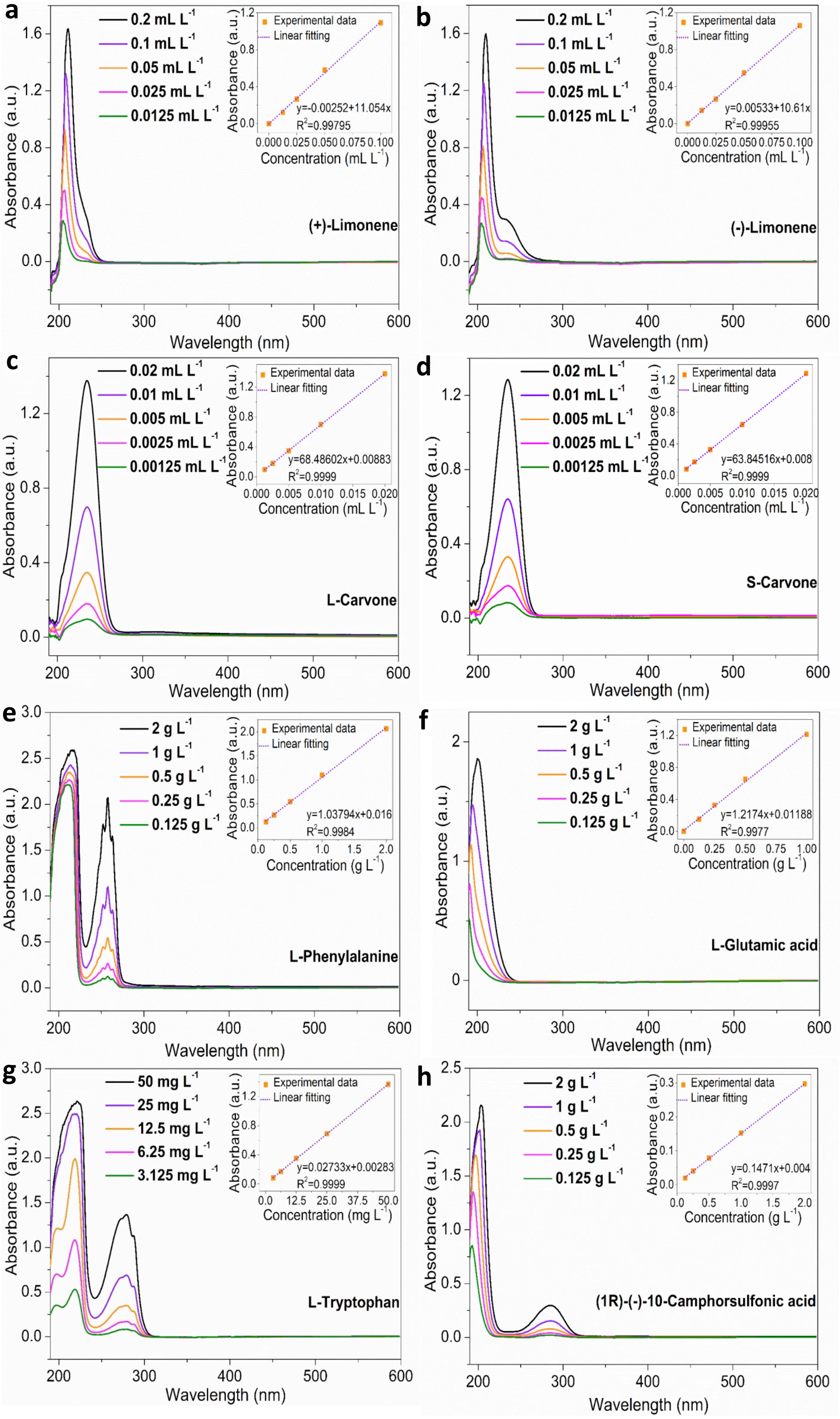

Supplementary Figure 21 | Standard curves of various enantiomers. The dotted violet lines denote the linear fitting results of solute absorbance. **a,** (+)-limonene. **b,** (-)-limonene. **c,** L-carvone. **d,** S-carvone. **e,** L-phenylalanine. **f,** L-glutamic acid. **g,** L-tryptophan. **h,** (1R)-(-)-10-camphorsulfonic acid.

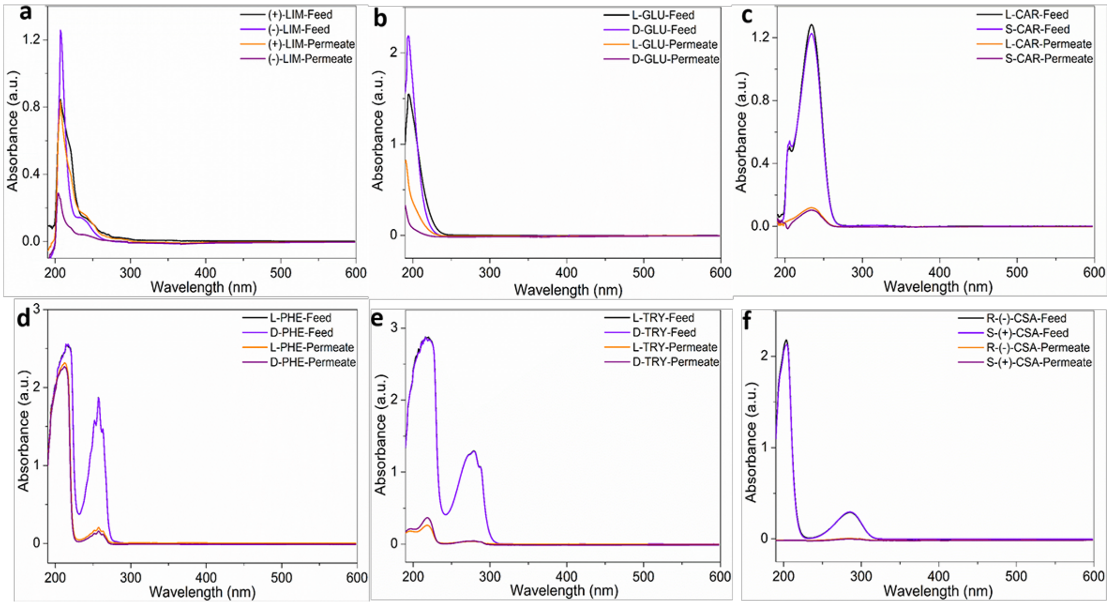

Supplementary Figure 22 | UV-Vis absorption spectra of enantiomers at feed and permeate compartments. a, (+)-LIM and (-)-LIM. **b,** L-GLU and D-GLU. **c,** L-CAR and S-CAR. **d,** L-PHE and D-PHE. **e,** L-TRY and D-TRY. **f,** R-(-)-CSA and S-(+)-CSA. LIM: limonene; GLU: glutamic acid; CAR: carvone; PHE: phenylalanine; TRY: tryptophan; CSA: camphorsulfonic acid.

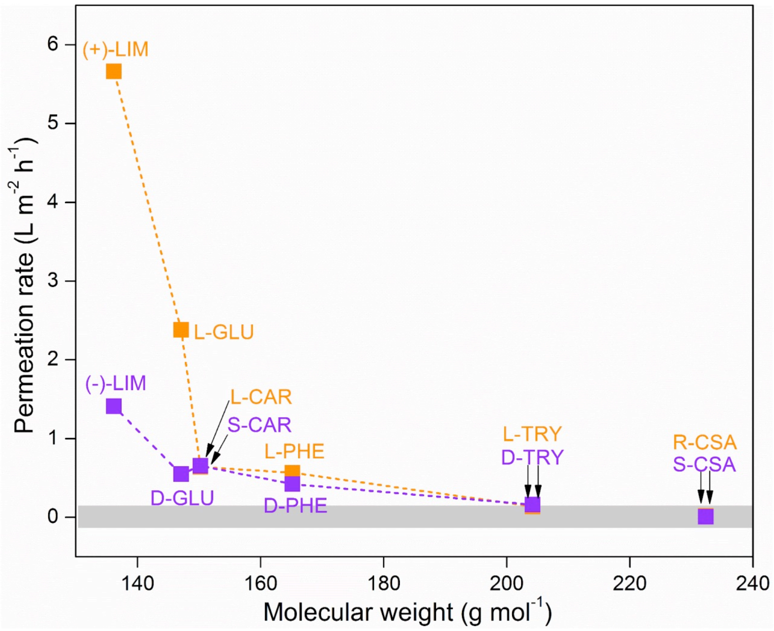

Supplementary Figure 23 | Permeation rates of enantiomers through GCN-CSA membrane. The dotted orange and violet lines denote the permeation rates of various pairs of enantiomers with different molecular weight.

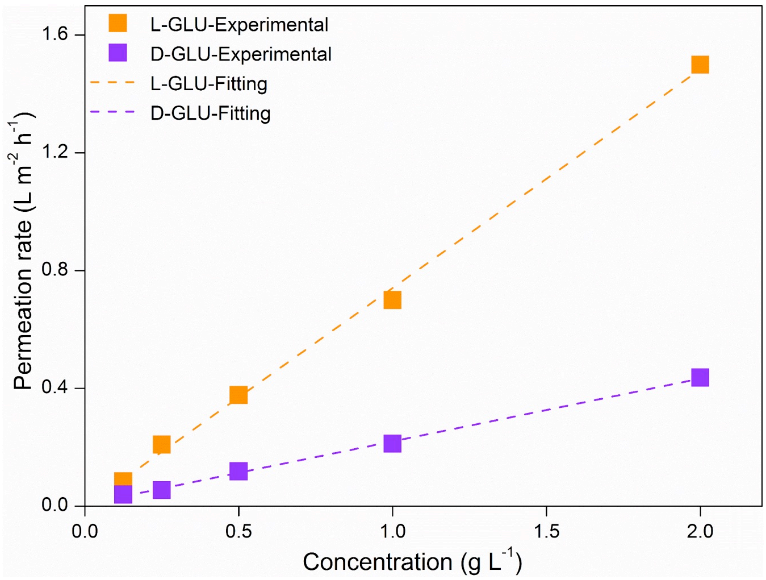

Supplementary Figure 24 | Permeation rates as a function of initial L/D-GLU concentrations. The dotted orange and violet lines are linear fitting results of permeation rates of L-GLU and D-GLU, respectively.

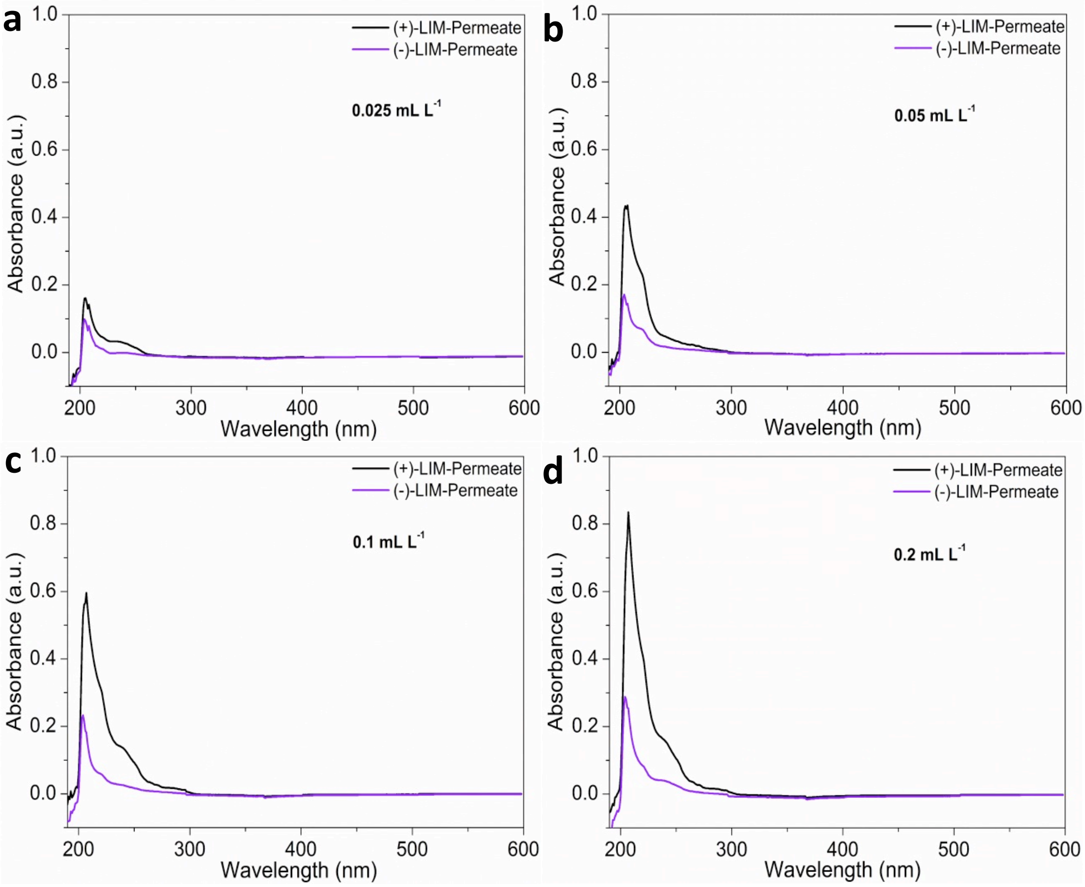

Supplementary Figure 25│ UV-Vis absorption spectra of (+)/(-)-LIM solutions at permeate compartments. (+)-LIM or (-)-LIM with varied concentrations are used as initial feed solutions over a period of 12 hrs. **a,** 0.025 mL L^{-1}. **b,** 0.05 mL L^{-1}. **c,** 0.1 mL L^{-1}. **d,** 0.2 mL L^{-1}.

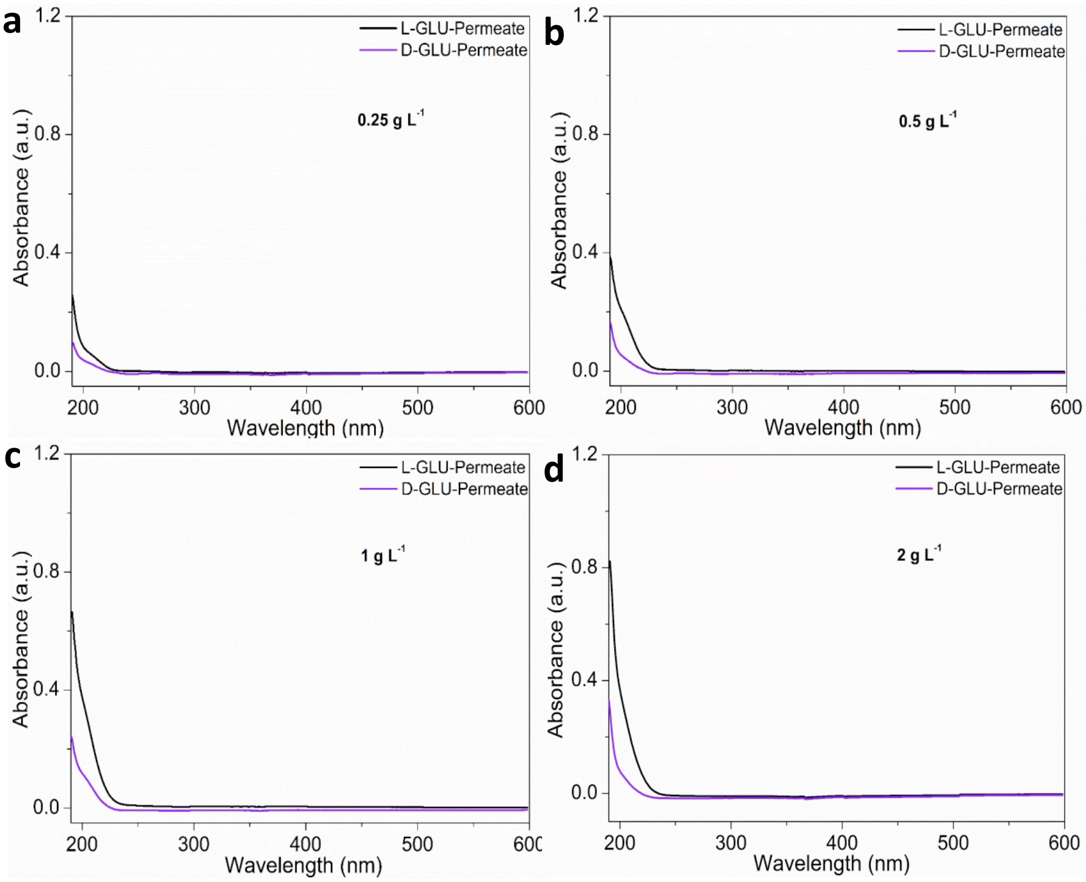

Supplementary Figure 26 | UV-Vis absorption spectra of L/D-GLU solutions at permeate compartments. L/D-GLU with varied concentrations are used as initial feed solutions over a period of 12 hrs. **a,** 0. 25 g L^{-1}. **b,** 0.5 g L^{-1}. **c,** 1 g L^{-1}. **d,** 2 g L^{-1}.

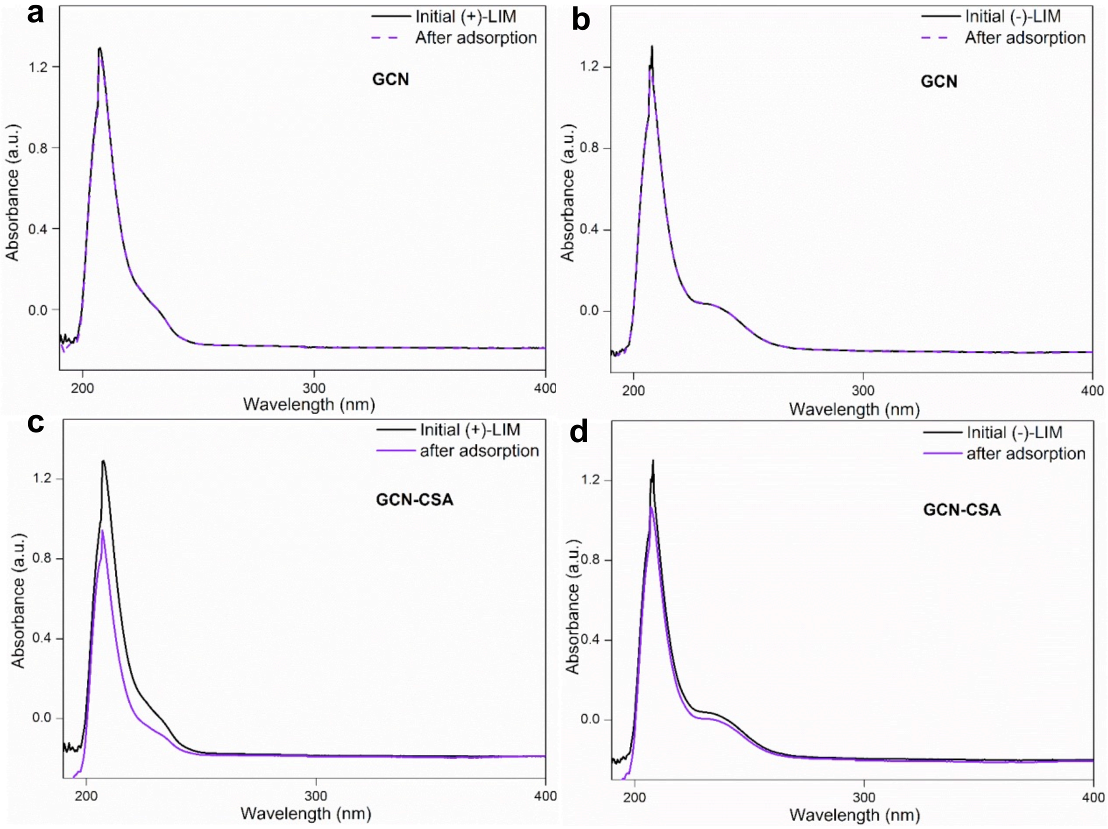

Supplementary Figure 27 | (+)/(-)-LIM adsorption of bulk GCN and GCN-CSA. **a,** (+)-LIM adsorption of GCN. **b,** (-)-LIM adsorption of GCN. **c,** (+)-LIM adsorption of GCN-CSA. **d,** (-)-LIM adsorption of GCN-CSA.

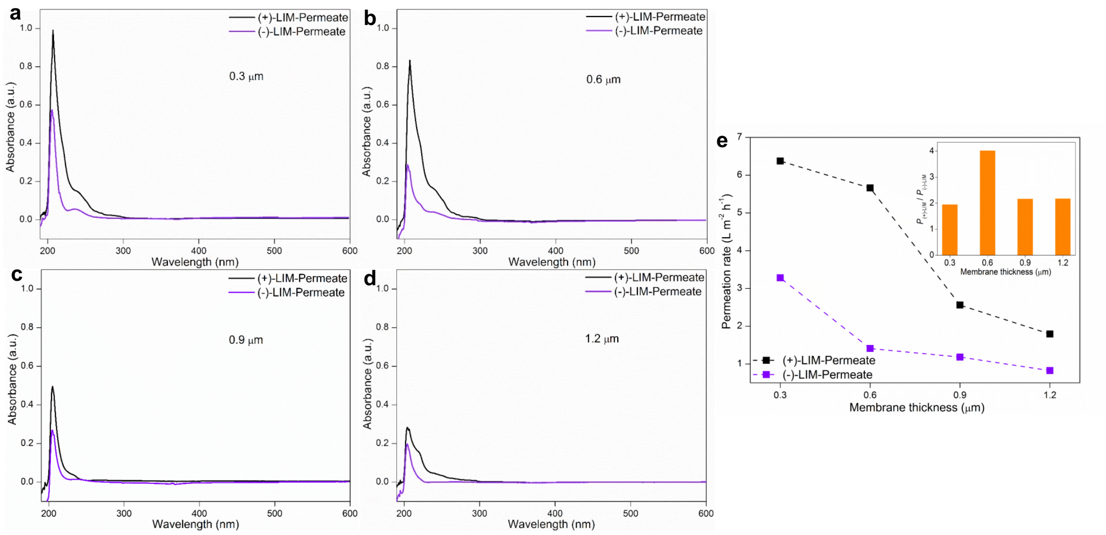

Supplementary Figure 28 | Thickness-dependent enantioselective permeation efficiency of (+)/(-)-LIM. a-d, UV-Vis absorption spectra of solutions at permeate compartments using 0.2 mL L^{-1} (+)-LIM or (-)-LIM as initial feed solutions and membranes with different thickness. **a**, 0.3 μm; **b**, 0.6 μm; **c**, 0.9 μm; **d**, 1.2 μm. **e**, Variation of permeation rates of (+)-LIM and (-)-LIM as a function of membrane thickness, the permeation rates are calculated according to Equation 3 in Methods section of main text. The dotted black and violet lines denote the variation of thickness-dependent permeation rates of (+)-LIM and (-)-LIM, respectively. Inset: the dependence of $P_{(+)\text{-LIM}}/P_{(-)\text{-LIM}}$ on membrane thickness, where P represents permeation rate. Higher $P_{(+)\text{-LIM}}/P_{(-)\text{-LIM}}$ value indicates greater difference of permeation rates between (+)-LIM and (-)-LIM.

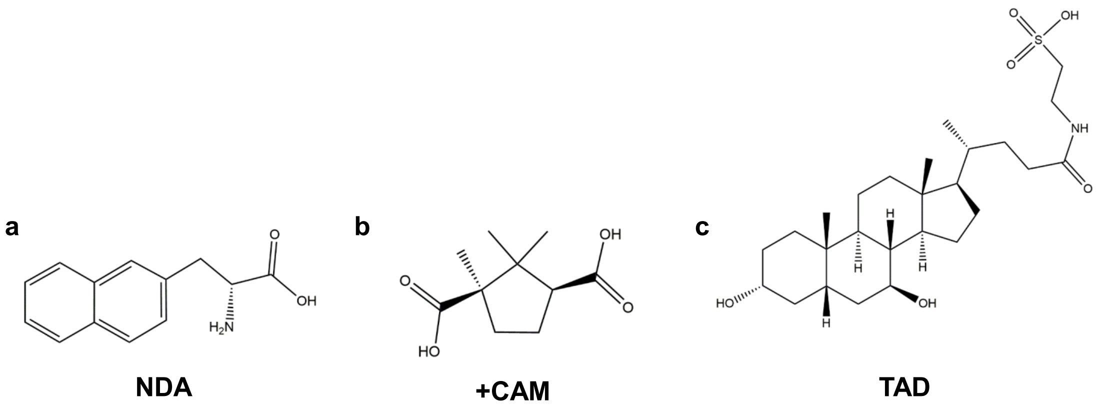

Supplementary Figure 29 | Molecular structures of different chiral intercalators. **a**, NDA. **b**, +CAM. **c**, TAD.

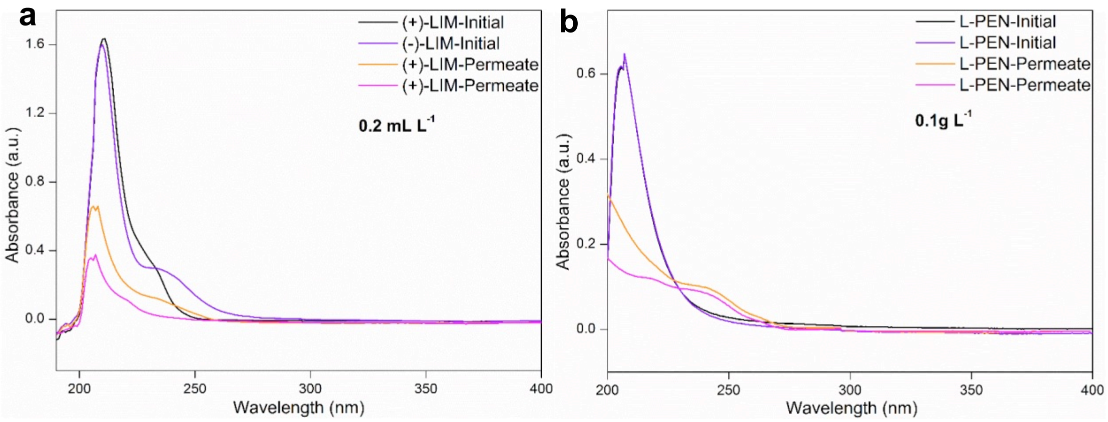

Supplementary Figure 30 | a, b, UV-Vis absorption spectra of initial and permeate solutions. **a**, 0.2 mL L^{-1} (+)/(-)-LIM. **b**, 0.1 g L^{-1} L/D-PEN (PEN: penicillamine).

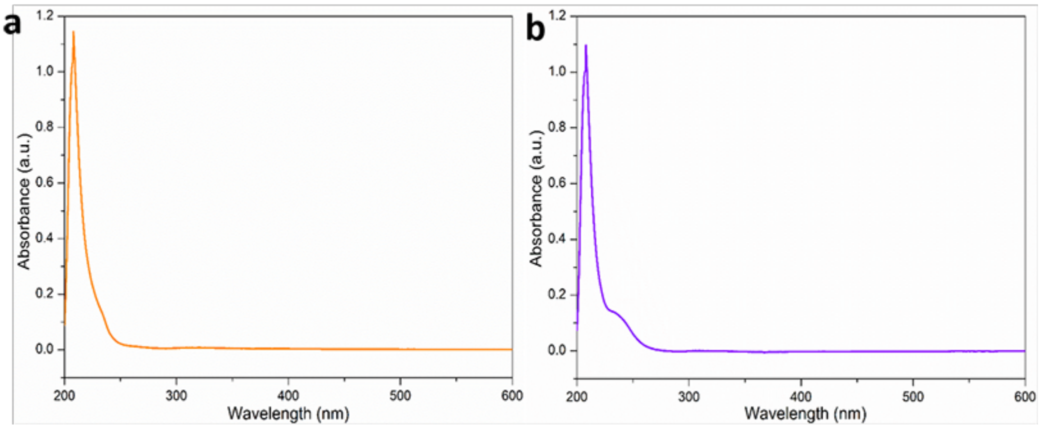

Supplementary Figure 31 | UV-Vis spectra of limonene racemate solution at two compartments. a, Permeate compartment. **b,** Feed compartment.

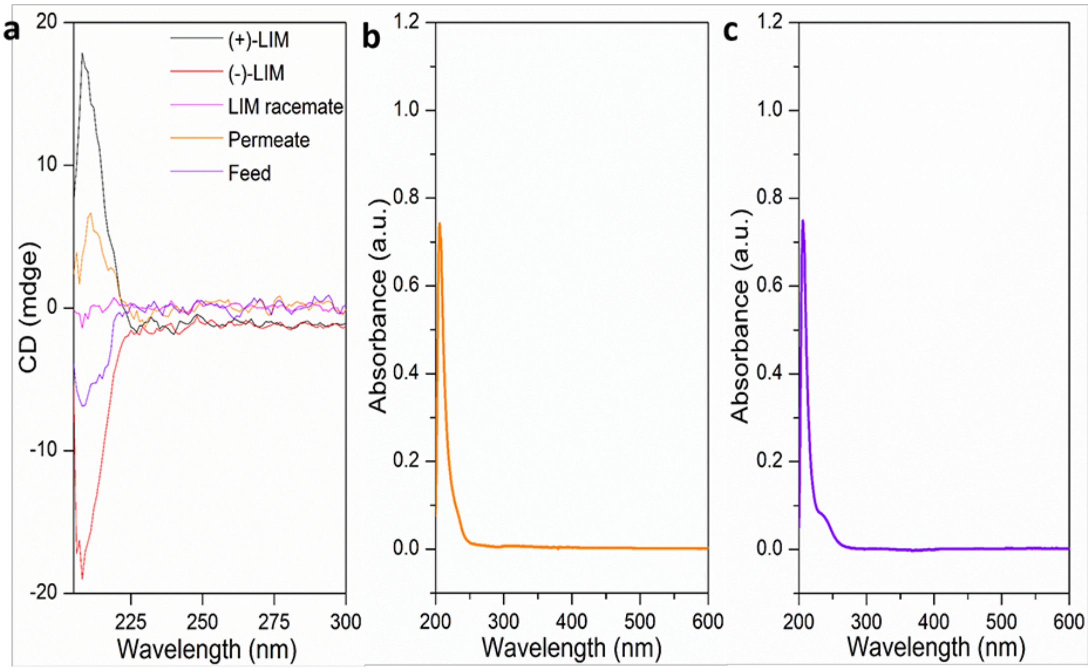

Supplementary Figure 32 | Analyses of LIM racemate permeation under reduced pressure. a, CD spectra. **b, c,** UV-Vis absorption spectra of solutions at permeate (**b**) and feed (**c**) compartments.

Supplementary Table 1 | Elemental analyses of GCN and GCN-SA.

Sample	C (wt%)	N (wt%)	S (wt%)	C/N (molar ratio)
GCN	34.95	60.74	/	0.67
GCN-SA	25.80	47.03	3.22	0.64

Supplementary Table 2 | Vaporizing pressure and ΔP of various solvents for permeability tests.

Solvent	Vaporizing pressure P_2 (kPa)	ΔP (kPa)
H_2O	2.338	97.662
MeOH	5.947	94.053
Dioxane	3.84	96.16
IPA	4.41	95.59
Ether	58.67	41.33
Cyclohexane	10.34	89.66

The vaporizing pressure (P_2) is obtained using the Langer's Handbook of Chemistry.

Supplementary Table 3 | Concentration, solvent and analysis method of solute solutions in permeation tests.

Solute	Concentration	Solvent	Analysis method
NaCl	1 mol L^{-1}	water	conductivity test
$MgCl_2$	1 mol L^{-1}	water	conductivity test
$K_3[Fe(CN)_6]$	1 g L^{-1}	water	UV-Vis spectra
sucrose	1 mol L^{-1}	water	TOC analysis [a]
AYR[b]	50 mg L^{-1}	water	UV-Vis spectra
MO[c]	50 mg L^{-1}	water	UV-Vis spectra
$Fe(phen)_3Cl_2$	50 mg L^{-1}	water	UV-Vis spectra
RhB[d]	50 mg L^{-1}	water	UV-Vis spectra
limonene	0.2 mL L^{-1}	ethanol	UV-Vis spectra
glutamic acid	2 g L^{-1}	water	UV-Vis spectra
carvone	0.02 mL L^{-1}	ethanol	UV-Vis spectra
phenylalanine	2 g L^{-1}	water	UV-Vis spectra
tryptophan	50 mg L^{-1}	water	UV-Vis spectra
camphorsulfonic acid	2 g L^{-1}	water	UV-Vis spectra

[a]TOC represents total organic carbon; [b]AYR: Alizarine Yellow R; [c]MO: Methyl Orange; [d]RhB: Rhodamine B.

Supplementary Table 4 | Hydrated radii of studied solutes for permeation tests.

Ion/Molecule	Hydrated radius (Å)	Ion/Molecule	Hydrated radius (Å)
K^+	3.31	(-)-limonene	4.33
		(+)-limonene	4.39
Cl^-	3.32	L-glutamic acid	3.99
Na^+	3.58	D-glutamic acid	4.02
Mg^{2+}	4.28	L-carvone	4.35
$Fe(CN)_6^{3-}$	4.75	S-carvone	4.41
sucrose	5.20	L-phenylalanine	4.32
AYR	5.34	D-phenylalanine	4.32
MO	5.47	R-camphorsulfonic acid	4.50
$Fe(phen)_3^{2+}$	6.46	S-camphorsulfonic acid	4.53
RhB	6.53	L-tryptophan	4.57
		D-tryptophan	4.58

Supplementary Table 5 | Concentration of feed and permeate compartments after 12 h permeation.

Solutes	Initial concentration	Feed	Permeate
NaCl	1 M	0.6994 M	0.2641 M
$MgCl_2$	1 M	0.7507 M	0.1945 M
$K_3[Fe(CN)_6]$	3.0373×10^{-3} M (1 g L^{-1})	2.527×10^{-3} M	3.523×10^{-4} M
sucrose	1 M	0.8995 M	0.0744 M
*AYR	1.7409×10^{-4} M (50 mg L^{-1})	1.6969×10^{-4} M	/
*MO	1.5275×10^{-4} M (50 mg L^{-1})	1.4958×10^{-4} M	/
*Fe(phen)	7.8442×10^{-5} M (50 mg L^{-1})	7.5700×10^{-5} M	/
*RhB	1.0438×10^{-4} M (50 mg L^{-1})	1.0115×10^{-4} M	/

*: the feed concentration of dye solutions keep unchanged after prolonging time from 12 hrs to three days.

Supplementary Table 6 | Elemental analyses of GCN and GCN-CSA.

Sample	C (wt%)	N (wt%)	S (wt%)	C/N (molar ratio)
GCN	34.95	60.74	/	0.67
GCN-CSA	33.60	41.16	2.852	0.65

Supplementary **Table 7 | Elemental analyses of GCN and GCN-CSA.**

Sample	C (wt%)	N (wt%)	S (wt%)	C/N (molar ratio of GCN)
GCN	34.95	60.74	/	0.67
GCN-CSA (1:1)	33.29	45.35	1.998	0.66
GCN-CSA (1:5)	33.60	41.16	2.852	0.65
GCN-CSA (1:10)	33.49	40.96	2.885	0.65

Supplementary Table 8 | Elemental analyses of GCN-CSA and AGCN-TAD.

Sample	C (wt%)	N (wt%)	S (wt%)	C/N (molar ratio of GCN)
GCN-CSA	33.60	41.16	2.852	0.65
AGCN-TAD	34.81	40.98	1.33	0.63

Note that carbon in CSA and the carbon and nitrogen in TDA were subtracted when calculating the C/N ratio. Considering the different molecular weights between CSA and TDA, the ratio of TAD to tri-s-triazine unit is determined to be 1:7.06, which is much lower than that in GCN-CSA sample (1:4.13).

Supplementary Note 1

GCN has been considered as a nitrogen-substituted graphene-like two-dimensional carbon material.[1] The structural unit of GCN was believed to be energetically favored tri-s-triazine.[2] With increasingly extended π-conjugated system from melamine, melem to melon, the solubility significantly decreases. And eventually, the polymerized GCN is insoluble in most solvents. Being similar with graphene and single-wall carbon nanotubes, GCN can also be dissolved in concentrated H_2SO_4, thus paving a convenient way for preparation of nanocomposites or assembly of macroscopic, ordered materials using a solution process.[3-5] In this work, the GCN-SA composite can be readily dispersed in water to form stable sol with higher concentration than GCN under sonication. The suspension can be stable for at least six months without any precipitation (Supplementary Figure 1). The high hydrophilicity of GCN-SA can be ascribed to the protonation of GCN sheets owing to the presence of sp^2 nitrogen atom.

The microstructure of GCN nanosheets characterized by TEM and AFM are shown in Supplementary Figures 2a-2c, which is consistent with the reported results.[6] As clearly revealed by TEM image in Supplementary Figure 2d, GCN-SA is also featured with a similar laminar texture with GCN. The AFM image further reveals that the thickness of the as-prepared GCN-SA nanosheets is 5-10 nm and the average lateral size is estimated to be ~150 nm, which is smaller than that of GCN (~280 nm). These results indicate that the protonation and anion intercalation did not evidently change the microstructure of pristine GCN. The increase in nanosheet thickness can be reasonably attributed to the aggregation of GCN-SA nanosheet during electrostatic interaction between protonated GCN layers and sulfate anion.

We collected XRD, XPS, FI-IR and elemental analysis data to understand the chemical structure of GCN-SA, besides TEM and AFM images. In comparison with exfoliated GCN nanosheets, the (002) diffraction peak at 27.6° got dramatically weaken in GCN-SA nanosheets, whereas new diffraction peaks at 6.28° and 12.42°

(001n) appeared, indicating a new phase with an interlayer spacing of 14.06 Å was produced (Supplementary Figure 3). Drying GCN-SA nanosheets at elevated temperature of 80 °C under vacuum did not change its structure, as XRD pattern remains intact. This confirmed that GCN-SA is thermally stable and the new diffraction is not coming from water layer incorporating in GCN layers. Furthermore, there was no swelling effect observed when re-immersing the dried GCN-SA into water for 3 days, which distinguishes it from the susceptive interlayer spacing (*d*) in water mediated GO membrane and partially exfoliated GGCN membrane.[7,8]

The XPS spectra of C 1*s*, N 1*s*, O 1*s* and S 2*p* of GCN and GCN-SA were recorded to gain insight into their chemical bonding environment (Supplementary Figure 4). For GCN, the main peak C1 centered at 288.4 eV was stemmed from sp^2 C atoms bonded to N (N-C=N) inside the aromatic structure; the other peak C2 at 284.8 eV was assigned to carbon impurities.[6] The four peaks of N 1*s* spectra confirmed the existence of C-N=C at 398.8 eV (N1), N-$(C)_3$ at 400.1 eV (N2), C-N-H at 401.4 eV (N3) and positive charge localization at 404.6 eV (N4), as marked in Supplementary Figure 5.[5,9] The C 1*s* and N 1*s* of GCN-SA spectra kept good consistence with that of GCN, indicating the retained chemical environment of C and N atoms. Note that the enhanced N3 signal (C-N-H) in GCN-SA was induced by protonation. A comparison between the S 2*p* spectra of GCN and GCN-SA clearly demonstrates the existence of sulfuric species in GCN-SA and the S 2*p* signal can be de-convoluted into $2p_{1/2}$ and $2p_{3/2}$ (peak area ≈ 1:2). The binding energy centered at 168.4 eV was indicative of SO_4^{2-},[10] which confirms the anion (SO_4^{2-}) intercalation in GCN-SA composites as surface attached SO_4^{2-} has been removed by a thorough washing procedure. In addition, the existence of oxygen with a binding energy centered at 532.8 eV was largely ascribed to the water absorbed in both GCN and GCN-SA, another peak at 531.3 eV of GCN-SA can be assigned to the oxygen of SO_4^{2-}.[10,11] Also, no other sulfide species associated with lower valence states of sulfur were detected,[11] thereby suggesting a redox-free process during GCN protonation and anion (SO_4^{2-}) intercalation in GCN-SA formation.

The characteristic tri-s-triazine ring sextant out of plane bending vibration at ~810 cm^{-1} was clearly identified for both GCN and GCN-SA in FT-IR spectra, as shown in Supplementary Figure 6.[12] A set of peaks located in the range of 1200-1700 cm^{-1} were indicative of C-N and C=N stretching vibrations in GCN and GCN-SA,[13] suggesting the retained tri-s-triazine-based framework of GCN in GCN-SA composite after protonation. Compared with GCN, the FT-IR spectra of GCN-SA shows evident peaks at 1114 and 981 cm^{-1} that can be assigned to S=O and S-O vibrations, respectively.[5] These data are in line with XPS analysis.

Elemental analyses data of GCN and GCN-SA are shown in Supplementary Table 1. Typically, C/N ratios of GCN range from 0.6-0.8, depending on the preparation procedures.[14] The C/N ratio of GCN-SA is slightly decreased in comparison with GCN itself, probably owing to partial hydrolysis in concentrated H_2SO_4 (98%).[15] Considering tri-s-triazine as the structural unit of GCN, the formula is regarded to be C_6N_8, because three units share each N of tertiary amine. Accordingly, the molar ratio of SO_4^{2-} to the unit is roughly evaluated to be 1:3.72.

Supplementary Note 2

Supplementary Figure 10 shows the standard curves of studied solutions permeation tests. Conductivities of NaCl and $MgCl_2$ solutions were measured using conductivity meter equipped with Pt black electrode. The electrode with cell constant of 9.65 was selected for determining the conductivity of solutions with high concentration. Another electrode with a cell constant of 1 was employed to monitor the conductivity of solutions with concentrations lower than 0.01 mol L^{-1}. The measured results were rechecked using TDS&EC water-quality testing meter with an available range of 0-9999 μS cm^{-1}. Note that the conductivity values were recorded at ~0 ^{o}C and the corresponding standard curves shown nonlinear tendency with changing concentration.

Supplementary Note 3

The UV-Vis absorption spectra of the feed and permeate solution for studied solutes at 12 hrs are shown in Supplementary Figure 11. There is no obvious permeation for large-sized molecules of AYR, MO, RhB and $Fe(phen)_3Cl_2$, even with prolonging permeation time to 72 hrs. Note that the rejection rates for these molecules shown in main text are in close proximity but not equal to 100%, as the spectra of solutions in permeate compartments give rise to almost negligible absorption peak.

In order to eliminate the effect of initial concentration difference of various solutes and to provide comparable average permeation rates among different solutes, all the initial concentration of tested solutes are set at 0.005 M (solvent: water).

Obviously, after taking average concentration difference into calculation according to Equation 3 in Methods section of main text, permeation rates of NaCl, $MgCl_2$, $K_3[Fe(CN)_6]$ and sucrose at the low initial concentration is similar with the previous results with high initial concentration (Supplementary Figure 12). Note that no UV-Vis absorption signals of solutions in permeate compartments were observed for AYR, MO, $[Fe(phen)_3]Cl_2$ and RhB during the measurements lasting for even three days, keeping good consistency with our previous results. The scattered points fall in the grey area of Supplementary Figure 12 represent the detection limit. The molecular sieving effects were evaluated again via plotting the permeation rate against the hydrated solute radius; the results are shown in Supplementary Figure 12. The data reveals the same cut-off behavior of the membrane.

Here also comes the concern about the adsorption effect of studied solutes that may exert impact on the permeation behavior of the solutes. To well address this issue, the concentration of both feed and permeate compartments are listed in Supplementary Table 5. For permeable solutes NaCl, $MgCl_2$, $K_3[Fe(CN)_6]$ and sucrose, the initial concentrations are high enough for saturated adsorption (provided the adsorption really takes place). As shown in Supplementary Table 5, the total number of molecules from both the feed and permeate compartments the

membrane is very close to that in the initial feed solutions, which indicates that the sieving performance is not falsified by adsorption effect. In this regard, we further crosschecked the possible adsorption via testing cycling performance of the membrane using fresh initial solution and pure solvent in both feed and permeate sides, respectively. The permeation performance is similar for three cycles, revealing the negligible adsorption effect.

As for the dye solutes with larger sizes, no obvious permeation was observed and negligible concentration decreasing was detected when using initial concentrations of 50 mg L^{-1} or even much higher initial concentrations of 0.005 M. This result suggests that dyes with initial concentrations of 50 mg L^{-1} are high enough for saturated adsorption. In addition, the feed compartments of these dye solutions exhibited no detectable UV-Vis spectra difference (that is, no detectable concentration decrease) when further prolonging time from 12 hrs to three days, which manifests 12 hrs is enough for saturated adsorption.

Supplementary Note 4

Along with the protonation of GCN, we attempted to substitute SO_4^{2-} accommodated in the interlayer space of GCN with CSA, but failed. Trials to direct intercalation of CSA into GCN using various solvents with assistance of sonication/refluxing also turned out to be unsuccessful. This is mainly ascribed to the lower acidity of CSA, and thus lower capability to protonate GCN.

With assistance of water, the mixture of GCN and CSA was subjected to ball milling, which provided continuous shear force and greatly promoted exfoliation and protonation, as well as the intercalation of the bulky CSA anion into the protonated GCN sheets. After thorough washing to remove excess and surface attached CSA, the yellowish powder was readily dispersing in water. Further centrifugation at 12000 rpm for 5min was applied to remove large aggregated nanoparticles. The GCN-CSA concentration of the supernatant is much higher than that of GCN, as shown in Supplementary Figure 13.

GCN-CSA shows similar microstructural feature with GCN-SA and GCN nanosheet, as shown in Supplementary Figure 14. The TEM image reveals a typical laminar texture of GCN-CSA nanosheets. The thickness of GCN-CSA measured by AFM falls in the range of 7.2-13.5 nm. The increase in nanosheet thickness can be reasonably attributed to the aggregation of GCN-CSA nanosheet during preparation and less-effective exfoliation by ball milling. The lateral size of GCN-CSA nanosheets is typically ~250 nm and similar with that of liquid-exfoliated GCN.

Similar to chemical structural analysis of GCN-SA, we collected XRD, XPS, FT-IR and elemental analysis data to understand the chemical structure of GCN-CSA, besides TEM and AFM images. Previous reports speculated that high-speed ball milling can damage the in-plane structure of GCN.[16] XRD patterns were recorded to check the structure of GCN-CSA, as shown in Supplementary Figure 15. As a reference, the suspension of GCN in water was also ball milled. The ball milling exerted negligible impact on the crystal structure of GCN, as the XRD patterns of

GCN treated with/without ball milling show the same diffraction (Supplementary Figure 15a). However, intensity of the (100) and (002) peaks of GCN-CSA shown in Supplementary Figure 15b experienced significantly decreased in comparison with that of bulk GCN, signifying a less periodic texture and effective exfoliation of bulk GCN into few layers.[17]

As shown in Supplementary Figure 16, the GCN nanosheets prepared by ball milling show unaltered chemical environment with respect to that of bulk GCN. For GCN-CSA, the ratio of sulfur to oxygen in GCN-CSA was in close proximity to 1:4, matching well with the molecular formula of CSA ($C_{10}H_{16}O_4S$) and therefore indicating the successful intercalation of CSA into GCN. The slightly increased ratio of C2/C1 signal further results from the carbon in CSA. Note that no evident shift of C1 and N1-N4 signals was observed for GCN-CSA with respect to that of GCN, verifying the retained chemical environment after CSA intercalation.

Compared with the FT-IR spectra of bulk GCN, we also detected the characteristic vibrations (~810 cm^{-1} for tri-s-triazine ring sextant out of plane bending vibration, 1200-1700 cm^{-1} for stretching vibrations of C-N and C=N) in GCN-CSA sample (Supplementary Figure 17). These findings suggested that the primary tri-s-triazine framework of GCN was not altered after CSA protonation. Moreover, the C=O stretching vibration at ~1734 cm^{-1} and SO_3^- vibration at 1043 cm^{-1} imply the existence of CSA in the studied sample.[18]

Elemental analyses data of GCN and GCN-CSA are shown in Supplementary Table 6. Similar with the analysis of GCN-SA, the molar ratio of SO_4^{2-} to the C_6N_8 unit is roughly evaluated to be 1:4.13. Note that carbon in CSA was subtracted when calculating the ratio.

Supplementary Note 5

As discussed in main text, the XRD patterns of GCN-SA membranes are identical with that of nanosheets. Similarly, GCN-CSA membranes also share identical XRD patterns with that of GCN-CSA nanosheets, as shown in Supplementary Figure 15 and Supplementary Figures 18a and 18b. The XRD pattern of GCN-CSA membrane without PTFE support is also recorded and shown in Supplementary Figure 18b. Compared with pristine bulk GCN, the diffraction peaks of GCN-CSA membrane without any support are significantly weakened, showing a less periodic texture. The evolution of diffraction peaks clearly indicate that the as-prepared GCN-CSA membrane is more prone to be amorphous, as stated in main text.

The SEM images (Fig. 2 in main text and Supplementary Figure 19) reveal that the as-prepared GCN, GCN-SA, GCN-CSA membranes are featured with smooth surface without identifiable cracks or pinholes. In order to measure the thickness of the membranes, we transferred the as-prepared membranes on MCE substrate to silicon wafer. Specifically, the membranes on MCE were placed into an acetone bath to quickly dissolve MCE, leaving freestanding membrane drifting in the bath. The freestanding membrane was further washed with fresh acetone for three times and methanol once to completely remove the MCE. Finally, the cleaned membrane was picked up and transferred onto silicon wafer (thickness: 0.5 mm). Upon solvent evaporation, the dried membranes can be adhered onto the silicon wafer without slip and warping.[19] As shown in Supplementary Figure 20, the as-prepared membranes are well aligned onto the silicon wafer. Typically, the thickness ranges from 500-700 nm for the GCN, GCN-SA and GCN-CSA membranes.

Supplementary Note 6

The UV-Vis absorption spectra of various enantiomers used for permeation tests have also been recorded and plotted in Supplementary Figure 21. Note that enantiomers of glutamic acid, phenylalanine, tryptophan and CSA exhibit identical spectra. Therefore, we only listed the spectra of one of the enantiomers. But the absorption spectra of the enantiomers limonene and carvone are slightly different in their absorption intensity, probably owing to the purity difference of the chemical reagents. To give accurate results for quantitative analyses of permeation tests, we list the UV-Vis absorption spectra of both L-carvone and S-carvone, as well (+)-limonene and (-)-limonene. The high R^2 values of all dotted lines marked in Supplementary Figure 21 indicate acceptable and reliable fitting results.

Note that there are shifts and disappearance of absorption peaks in Supplementary Figures 21a, 21b and 21f, which are ascribed to the enhanced effect from solvent peak with decreasing concentrations. For all cases, favorable linear dependence of absorbance on concentration of different solutes are observed, the absorption peak locations of solutes at different concentration are determined based on the sample with highest concentration within our test range. For example, in Supplementary Figure 21a, the absorbance peak location is 211 nm for 0.2 $mL \cdot L^{-1}$ (+)-LIM, this location is also applicable for (+)-LIM with other concentrations (0.1, 0.05, 0.025 and 0.0125 $mL \cdot L^{-1}$) to determine the absorbance values, which is evidenced by the linear calibration curve.

Supplementary Note 7

Various enantiomers with different sizes and polarities were selected in this work to evaluate the permeation differences. Permeation tests of each pair of enantiomers were carried out separately over the same GCN-CSA membrane. The chemical stability of GCN-CSA membrane on PTFE substrate is excellent against ethanol, rendering facile cleaning and reusing of the membrane for checking repeatability. As shown in Supplementary Figure 22, enantioselectivity was only detected towards the enantiomers of limonene and glutamic acid. For other enantiomers, they showed the same permeation behaviors and thus no applicable enantioselectivity. Furthermore, the permeation rate decreased dramatically with increasing molecular weight over a period of 12 hrs.

The permeation rates of each pair of enantiomers calculated according to Equation 3 in Methods section of main text are displayed in Supplementary Figure 23, which are used for the calculation of the average permeation rates (Fig. 4a in main text) using the following Supplementary Equation 1:

$$\text{Average permeation rate} = (P_x + P_y)/2 \tag{1}$$

where P_x and P_y are permeation rates (considering average concentration difference) of the two enantiomers with the same molecular weight, respectively. For example, P_x is the permeation rate of (+)-LIM and P_y is the permeation rate of (-)-LIM provided in Supplementary Figure 23. Accordingly, the average rejection rates (inset of Fig. 4a in main text) are calculated based on Fig. 4b in main text and the following Supplementary Equation 2:

$$\text{Average rejection rate} = (R_x + R_y)/2 \tag{2}$$

where R_x and R_y are rejection rates of the two enantiomers with the same molecular weight, respectively. For example, R_x is the rejection rate of (+)-LIM and R_y is the rejection rate of (-)-LIM provided in Fig. 4b in main text.

The enantiomers of limonene and glutamic acid were selected to further investigate the permeation behaviors against different initial concentrations. As shown in Fig. 4(c) in main text and Supplementary Figure 24, the permeation rates were proportional with the initial concentration of solutions at feed compartments. The permeation rates were calculated using Equation 3 in Methods section of main text, based on the UV-Vis absorption intensity of solutions at permeate compartments, as shown in Supplementary Figures 25 and 26.

Supplementary Note 8

The adsorption experiments are conducted as follows: powder samples of 100 mg (GCN and GCN-CSA) were dispersed into 20 mL 0.1 mL L^{-1} (+)-LIM or (-)-LIM solution. We use 100 mg powder sample instead of membrane for adsorption test, because sample amount on membrane is very low so that the adsorption effect is hardly observed. After free standing for 12 hrs (the duration time is enough for adsorption-desorption equilibrium), the suspensions were centrifuged at 10000 rpm for 5min to obtain the supernatant, which were then subjected to UV-Vis absorption test. The results are shown in Supplementary Figure 27.

Supplementary Note 9

The permeation of limonene racemate solution composed of the mixture of (+)/(-)-LIM with an equal concentration of 0.1 mL L^{-1} was monitored by CD spectra. The feed and permeate compartments were filled with 0.1 mL L^{-1} limonene racemate in ethanol and pure ethanol (typically 5 mL in each compartment), respectively. The separation efficiency of limonene racemate was determined via detecting the CD signal variation of each compartment. As shown in Fig. 4 in main text, the CD signal of both feed and permeate solutions change in opposite directions with prolonging operation time, indicating that the (+)-LIM excess at permeate compartment and (-)-LIM excess at feed compartment, respectively. The solution at two compartments after 24 hrs permeation were analyzed by UV-Vis absorption spectra (Supplementary Figure 31), which determines the total concentration of both (+)-LIM and (-)-LIM according to their standard curves. Combining both UV-Vis and CD data, we are able to calculate the enantiomeric excess (*ee*) value.

The *ee* value of 89% after 24 hrs permeation of LIM racemate motivated us to further explore the possibility for realizing high selectivity within short operation time. Using a similar operation system under reduced pressure as shown in Supplementary Figure 8a, by replacing air pump with peristaltic pump (Leadfluid, BT 100L, China) operating at full speed, the solution at feed compartment (5 mL) can pass through GCN-CSA membrane within 3 min. The CD and UV-Vis spectra of solutions were recorded, as shown Supplementary Figure 32. The *ee* value was then calculated to be 75% accordingly, lower than that of isobaric permeation test as discussed above. However, the short operation time is believed to be more suitable for practical applications, we will direct our research focus on the improvement of *ee* value in future work.

Supplementary Methods

Actually, sulfonic acids are of stronger acidity in a diverse class of organic acids, which motivated us to prepare GCN-CSA composite using CSA that contains sulfonate. It was found that CSA can protonate GCN and further intercalate into the interlayer, showing similar functionalization mechanism with SA. The GCN-CSA membrane shows highly enantioselective permeation capability. We also selected TAD with sulfonate to tailor the interlayer spacing and chemical environment of GCN (Supplementary Figure 29c). However, the ball-milling assisted sonication (procedure for GCN-CSA) gave rise to low intercalation efficiency in GCN-TAD, probably due to its stronger steric hindrance of TAD than that of CSA. Here we adopt a modified experimental procedure that started from the use of acidified GCN as raw material (abbreviated as AGCN, see experimental procedures below).

Experimental Procedures for AGCN-TAD: 1) AGCN preparation: 5 mL of GCN-SA solution was heated at 70 °C for 1h, upon which 70 mL deionized water was injected and 7.5 g NH_4Cl powder was added. The suspension was subjected to stirring for 1 h and allowed to stand for another 0.5 h. And then the hot filtration is applied to obtain colorless filtrate, which was quickly transferred to ice-water bath and stirred for 1 h to obtain white suspension. Subsequently, the suspension was centrifuged at 8000 rpm for 5 min. The as-obtained precipitation was washed with H_2O and ethanol for 3 times, followed by drying at 60 °C under vacuum. Finally, the acidized GCN powder was obtained and denoted as AGCN.[15]

2) AGCN-TAD preparation: AGCN of 10 mg was dispersed into 0.2 M hydrochloric DMF solution (10 mL) and sonicated for 2 hrs to obtain colorless solution. Excess amount of TAD (100 mg) was added into and quickly dissolved in the solution, which was then stirred for 1 h at room temperature. Anti-solvent $CHCl_3$ (20 mL) was selected and added into the solution, the as-obtained colloidal precipitation was washed with $CHCl_3$ for 3 times and re-dispersed into 10 mL H_2O,

followed by sonication for 3 hrs to obtain highly dispersed AGCN-TAD without obvious precipitation after free standing for one month.

Here we also emphasize that GCN in organic chiral acids can only form suspensions with limited concentration during sample preparation process due to larger organic groups, which differs from the case in homogeneous GCN-SA solution. The non-homogeneous system precludes high intercalation efficiency to some extent, thereby leading to lower precision in tailoring interlayer distance of GCN. Nonetheless, the above results and discussion already indicate the universal strategy for GCN functionalization with assistance of either inorganic or organic acids. Our future work will focus on the use of other appropriate chiral acids for highly efficient enantioselective permeation.

Supplementary References

1. Ong, W. J. *et al.* Graphitic carbon nitride (g-C_3N_4)-based photocatalysts for artificial photosynthesis and environmental remediation: are we a step closer to achieving sustainability? *Chem. Rev.* **116**, 7159-7329 (2016).
2. Zheng, Y. *et al.* Graphitic carbon nitride polymers toward sustainable photoredox catalysis. *Angew. Chem. Int. Ed.* **54**, 12868-12884 (2015).
3. Behabtu, N. *et al.* Spontaneous high-concentration dispersions and liquid crystals of graphene. *Nat. Nanotechnol.* **5**, 406-411 (2010).
4. Davis, V. A. *et al.* True solutions of single-walled carbon nanotubes for assembly into macroscopic materials. *Nat. Nanotechnol.* **4**, 830-834 (2009).
5. Zhou, Z. X. *et al.* Dissolution and liquid crystals phase of 2D polymeric carbon nitride. *J. Am. Chem. Soc.* **137**, 2179-2182 (2015).
6. Zhang, X. D. *et al.* Enhanced photoresponsive ultrathin graphitic-phase C_3N_4 nanosheets for bioimaging. *J. Am. Chem. Soc.* **135**, 18-21 (2013).
7. Cerveny, S. *et al.* Dynamics of water intercalated in graphite oxide. *J. Phys. Chem. C* **114**, 2604-2612 (2010).
8. Wang, Y. J. *et al.* Water transport with ultralow friction through partially exfoliated g-C_3N_4 nanosheet membranes with self-supporting spacers. *Angew. Chem. Int. Ed.* **56**, 8974-8980 (2017).
9. Lin, L. H. *et al.* Tri-s-triazine-based crystalline graphitic carbon nitrides for highly efficient hydrogen evolution photocatalysis. *ACS Catal.* **6**, 3921-3931 (2016).
10. Terlingen, J. G. A., Feijen, J. & Hoffman, A. S. Immobilization of surface active compounds on polymer supports using glow discharge processes. *J. Colloid Interf. Sci.* **155**, 55-65 (1993).
11. Wang, K. *et al.* Sulfur-doped g-C_3N_4 with enhanced photocatalytic CO_2-reduction performance. *Appl. Catal. B-Environ.* **176**, 44-52 (2015).
12. Zhang, Y. Y. *et al.* Reversible assembly of graphitic carbon nitride 3D network for highly selective dyes absorption and regeneration. *ACS Nano* **10**, 9036-9043 (2016).

13. Xu, J. *et al.* Chemical exfoliation of graphitic carbon nitride for efficient heterogeneous photocatalysis. *J. Mater. Chem. A* **1**, 14766-14772 (2013).
14. Thomas, A. *et al.* Graphitic carbon nitride materials: variation of structure and morphology and their use as metal-free catalysts. *J. Mater. Chem.* **18**, 4893-4908 (2008).
15. Du, X. R. *et al.* A scalable chemical route to soluble acidified graphitic carbon nitride: an ideal precursor for isolated ultrathin g-C_3N_4 nanosheets. *Nanoscale* **7**, 8701-8706 (2015).
16. Ji, J. J. *et al.* Simultaneous noncovalent modification and exfoliation of 2D carbon nitride for enhanced electrochemiluminescent biosensing. *J. Am. Chem. Soc.* **139**, 11698-11701 (2017).
17. Niu, Peng. *et al.* Graphene-like carbon nitride nanosheets for improved photocatalytic activities. *Adv. Funct. Mater.* **22**, 4763-4770 (2012).
18. Hou, X. D. *et al.* Conductive and chiral polymer-modified metal-organic framework for enantioselective adsorption and sensing. *ACS Appl. Mater. Inter.* **10**, 26365−26371 (2018).
19. Wu, Z. C. *et al.* Transparent, conductive carbon nanotube films. *Science* **305**, 1273-1276 (2004).
